# Transient ST-segment elevation following coronary artery bypass grafting in a patient with immune thrombocytopenia treated with romiplostim: a case report

**DOI:** 10.1186/s43044-026-00776-x

**Published:** 2026-09-10

**Authors:** Zakiur Rehman Ansari, Zainul Abedein Hamdulay, Azizullah Khan, Sanjesh Jain, Meher Hamdulay, Aziz Kothawala

**Affiliations:** 1 Department of Clinical Research in Cardiovascular Surgery and Critical Care Medicine, Masina Heart Institute & GHC Hospitals, Mumbai, Maharashtra India; 2 Department of Cardiothoracic Surgery, Masina Heart Institute & GHC Hospitals, Mumbai, Maharashtra India; 3 Department of Critical Care Medicine, Masina Heart Institute & GHC Hospitals, Mumbai, Maharashtra India; 4 Department of Cardiac Anaesthesia, Masina Heart Institute & GHC Hospitals, Mumbai, Maharashtra India; 5 Department of Cardiology, Masina Heart Institute & GHC Hospitals, Mumbai, Maharashtra India

**Keywords:** Immune thrombocytopenia, Coronary artery bypass grafting, ST-segment elevation, Postoperative pericardial inflammation, Romiplostim, Pericarditis, Cardiac surgery complications

## Abstract

**Introduction:**

ST-segment elevation following coronary artery bypass grafting (CABG) is a clinically important postoperative finding that may indicate perioperative myocardial infarction, early graft failure, or acute coronary compromise. However, non-ischemic postoperative processes may produce similar electrocardiographic abnormalities, creating diagnostic uncertainty, particularly in clinically stable patients. Patients with immune thrombocytopenia (ITP) undergoing cardiac surgery present an additional therapeutic challenge because of the competing risks of bleeding and thrombosis. Thrombopoietin receptor agonists (TPO-RAs), such as romiplostim, may be used to increase platelet counts before invasive procedures, but their use requires careful assessment in patients with concomitant cardiovascular disease.

**Case presentation:**

A 47-year-old man with chronic ITP and multivessel coronary artery disease underwent elective CABG following preoperative administration of romiplostim for platelet optimization. Forty-eight hours after receiving romiplostim, his platelet count increased from 57 × 10⁹/L to 125 × 10⁹/L. Four-vessel CABG was performed, with a pedicled left internal mammary artery (LIMA) grafted to the left anterior descending artery (LAD) and a right internal mammary artery (RIMA) used as a Y-graft arising from the LIMA and directed sequentially to the diagonal branch, ramus intermedius, and posterior descending artery (PDA). The operation was initially attempted off-pump but was converted to cardiopulmonary bypass because of challenging coronary anatomy and inadequate target-vessel exposure. On postoperative day one, routine electrocardiography demonstrated new ST-segment elevation in leads II, III, and aVF, accompanied by PR-segment depression. A pericardial friction rub was documented. The patient remained clinically asymptomatic and hemodynamically stable. Transthoracic echocardiography showed preserved left ventricular systolic function without new regional wall motion abnormalities or pericardial effusion. High-sensitivity cardiac troponin I (hs-cTnI) was 0.9 ng/mL on postoperative day one and declined to 0.3 ng/mL on postoperative day three (laboratory reference range, 0–0.6 ng/mL). C-reactive protein and erythrocyte sedimentation rate were elevated to 120 mg/L and 68 mm/h, respectively; preoperative inflammatory marker values were unavailable.Given the absence of clinical or echocardiographic evidence of ongoing ischemia, declining cardiac biomarkers, preserved ventricular function, and the additional procedural bleeding risk associated with ITP, immediate coronary angiography was deferred. The patient was managed with close monitoring, aspirin, prophylactic low-molecular-weight heparin, indomethacin, and intravenous nicorandil during the initial assessment. The ST-segment elevation resolved by postoperative day three without clinical evidence of ongoing myocardial ischemia.

**Conclusion:**

Transient ST-segment elevation after CABG requires systematic evaluation for both ischemic and non-ischemic causes. In this patient, the combination of inferior ST-segment elevation, PR-segment depression, a documented pericardial friction rub, preserved ventricular function, absence of regional wall motion abnormalities, declining hs-cTnI, elevated inflammatory markers, and spontaneous ECG resolution favored an early postoperative inflammatory process. Romiplostim should be regarded as part of the patient’s overall thrombotic-risk profile rather than as a proven direct cause of the transient ECG abnormality.

## Introduction

ST-segment elevation following coronary artery bypass grafting (CABG) is an important postoperative electrocardiographic finding that warrants careful evaluation. In the early postoperative period, ST-segment elevation may indicate potentially serious ischemic complications, including perioperative myocardial infarction, acute graft failure, anastomotic complications, or native coronary artery compromise [1]. Prompt recognition is therefore important because delayed identification of significant graft or coronary pathology may result in irreversible myocardial injury.

Interpretation of postoperative myocardial injury is challenging because cardiac biomarker elevation may occur as a consequence of surgical myocardial manipulation, cardiopulmonary bypass, cardioplegic arrest, and ischemia-reperfusion injury. Accordingly, assessment for perioperative myocardial infarction should integrate cardiac biomarker kinetics with new electrocardiographic abnormalities, imaging evidence of myocardial dysfunction, clinical findings, and angiographic assessment when clinically indicated [2]. Thus, isolated postoperative ST-segment elevation should not be interpreted independently but should be correlated with the patient’s clinical status, serial biomarkers, echocardiographic findings, and hemodynamic course.

Non-ischemic processes may also produce postoperative ST-segment elevation. Pericardial inflammation following cardiac surgery can produce ST-segment elevation and PR-segment changes and may clinically mimic acute coronary ischemia [3]. Coronary vasospasm, myocardial edema, and transient myocardial stunning are additional potential explanations for postoperative electrocardiographic abnormalities [1].

Immune thrombocytopenia (ITP) creates an additional challenge in patients requiring invasive cardiovascular procedures because thrombocytopenia increases bleeding risk while coronary disease may simultaneously necessitate antiplatelet and anticoagulant therapy. Previous reports describing invasive cardiac treatment in patients with ITP emphasize the need for individualized assessment of platelet counts, bleeding risk, antithrombotic treatment, and the urgency of coronary intervention [4].

Thrombopoietin receptor agonists (TPO-RAs), including romiplostim, are used to increase platelet counts in patients with chronic ITP, particularly when an invasive procedure is anticipated. Although these agents can facilitate procedural platelet optimization, thromboembolic events have been reported, and their use warrants consideration of the patient’s overall thrombotic risk, particularly in those with established cardiovascular disease [5, 6].

Pericarditis is diagnosed on the basis of an integrated clinical assessment rather than ECG findings alone. Typical features include characteristic chest pain, pericardial friction rub, characteristic ECG changes, and/or new or worsening pericardial effusion, with inflammatory markers providing supportive rather than specific evidence [7]. This distinction is particularly relevant after cardiac surgery, where systemic inflammatory responses are common.

Although postoperative ST-segment elevation after CABG is well recognized, limited clinical information is available regarding its evaluation in patients with ITP receiving preoperative romiplostim. Such patients present a particularly complex balance between ischemic risk, thrombotic risk, bleeding risk, and the hazards of invasive diagnostic procedures.

We report a case of transient inferior ST-segment elevation following CABG in a patient with chronic ITP who received preoperative romiplostim. The case illustrates the importance of integrating electrocardiographic findings with serial cardiac biomarkers, echocardiography, inflammatory findings, surgical anatomy, and individualized procedural risk when distinguishing possible ischemic complications from postoperative inflammatory changes.

## Case presentation

### Patient information

A 47-year-old man presented with a three-month history of exertional chest discomfort consistent with Canadian Cardiovascular Society class II angina. He had a known history of chronic immune thrombocytopenia diagnosed five years earlier and was under regular hematological follow-up. There was no history of diabetes mellitus, chronic kidney disease, previous cerebrovascular disease, prior myocardial infarction, or significant spontaneous bleeding episodes. The patient was referred for surgical myocardial revascularization following evaluation for symptomatic multivessel coronary artery disease.

### Diagnostic assessment

Coronary angiography demonstrated significant triple-vessel coronary artery disease involving the left anterior descending artery (LAD), right coronary artery (RCA), and obtuse marginal branch. In view of the extent of coronary disease and the patient’s symptomatic status, elective CABG was planned.

The preoperative platelet count was 57 × 10⁹/L. Considering the underlying ITP and the anticipated perioperative platelet consumption associated with major cardiac surgery and cardiopulmonary bypass, preoperative platelet optimization was considered necessary.

The patient received romiplostim 250 µg subcutaneously 48 h before the planned surgical procedure. His platelet count subsequently increased to 125 × 10⁹/L, permitting elective surgical revascularization.

### Surgical procedure

The patient underwent four-vessel coronary artery bypass grafting under general anesthesia. A pedicled left internal mammary artery (LIMA) was grafted to the left anterior descending artery (LAD), while the right internal mammary artery (RIMA) was used as a Y-graft arising from the LIMA and directed sequentially to the diagonal branch, ramus intermedius, and posterior descending artery (PDA). Thus, the RCA territory was revascularized through the PDA graft.

The operation was initially attempted under off-pump conditions. However, unfavorable coronary anatomy, including a deeply intramyocardial LAD and difficulty in adequately identifying and exposing the distal PDA and posterolateral ventricular (PLV) coronary targets, made precise and safe distal coronary anastomosis technically challenging under off-pump conditions. Conversion to cardiopulmonary bypass was therefore undertaken to facilitate optimal myocardial stabilization, coronary exposure, and accurate graft anastomosis. The procedure was subsequently completed without immediate surgical complications.

The early postoperative period was initially stable, with satisfactory hemodynamic recovery and no evidence of excessive bleeding.

### Postoperative electrocardiographic finding

**Fig. 1:**
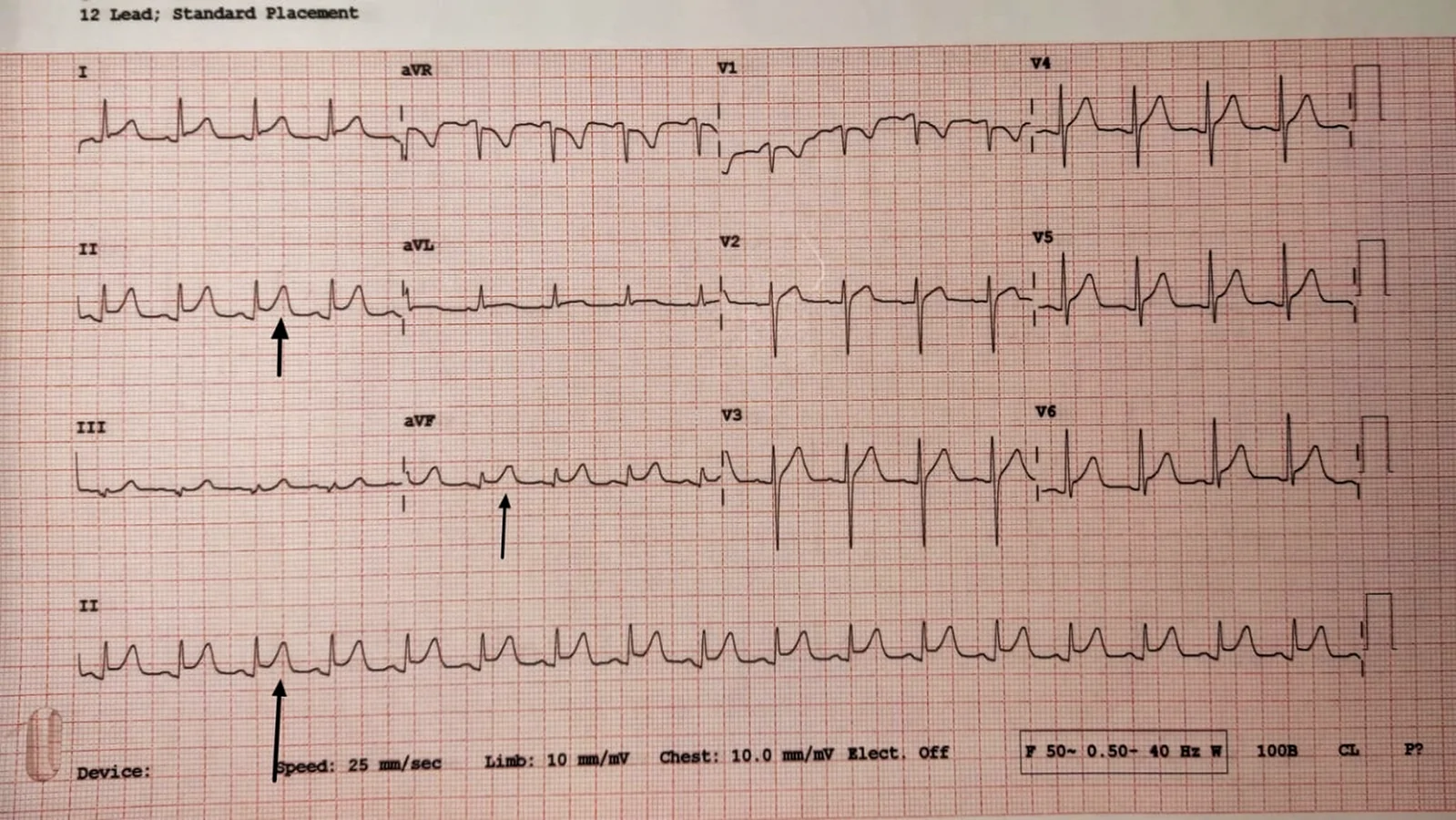
Twelve-lead electrocardiogram obtained on postoperative day one demonstrating ST-segment elevation in the inferior leads (II, III, and aVF) (arrows)

On postoperative day one, routine 12-lead electrocardiography demonstrated new ST-segment elevation involving leads II, III, and aVF (Fig. 1). The ECG abnormality was accompanied by PR-segment depression. No reciprocal ST-segment depression was observed in the anterior leads.

At the time of the ECG abnormality, the patient had no chest pain and remained hemodynamically stable. There was no requirement for escalation of vasoactive support and no clinical evidence of low cardiac output syndrome. A pericardial friction rub was documented on clinical examination.

Because post-sternotomy analgesia may reduce or mask the perception of chest pain, the absence of chest discomfort was not considered sufficient to exclude myocardial ischemia or pericardial inflammation. Further investigations were therefore undertaken to differentiate ischemic from inflammatory causes.

### Investigations

Following detection of inferior ST-segment elevation, serial clinical, biochemical, electrocardiographic, and echocardiographic assessments were performed.

C-reactive protein was 120 mg/L and erythrocyte sedimentation rate was 68 mm/h. These inflammatory markers were elevated; however, preoperative CRP and ESR values had not been obtained and were therefore unavailable for comparison. The inflammatory marker elevation was consequently interpreted as supportive of postoperative inflammation but not as diagnostic of pericarditis. The postoperative platelet count was 78 × 10⁹/L on postoperative day one. Subsequent platelet counts were 75 × 10⁹/L on postoperative day two, 81 × 10⁹/L on postoperative day three, and 85 × 10⁹/L on postoperative day four.

**Fig. 2:**
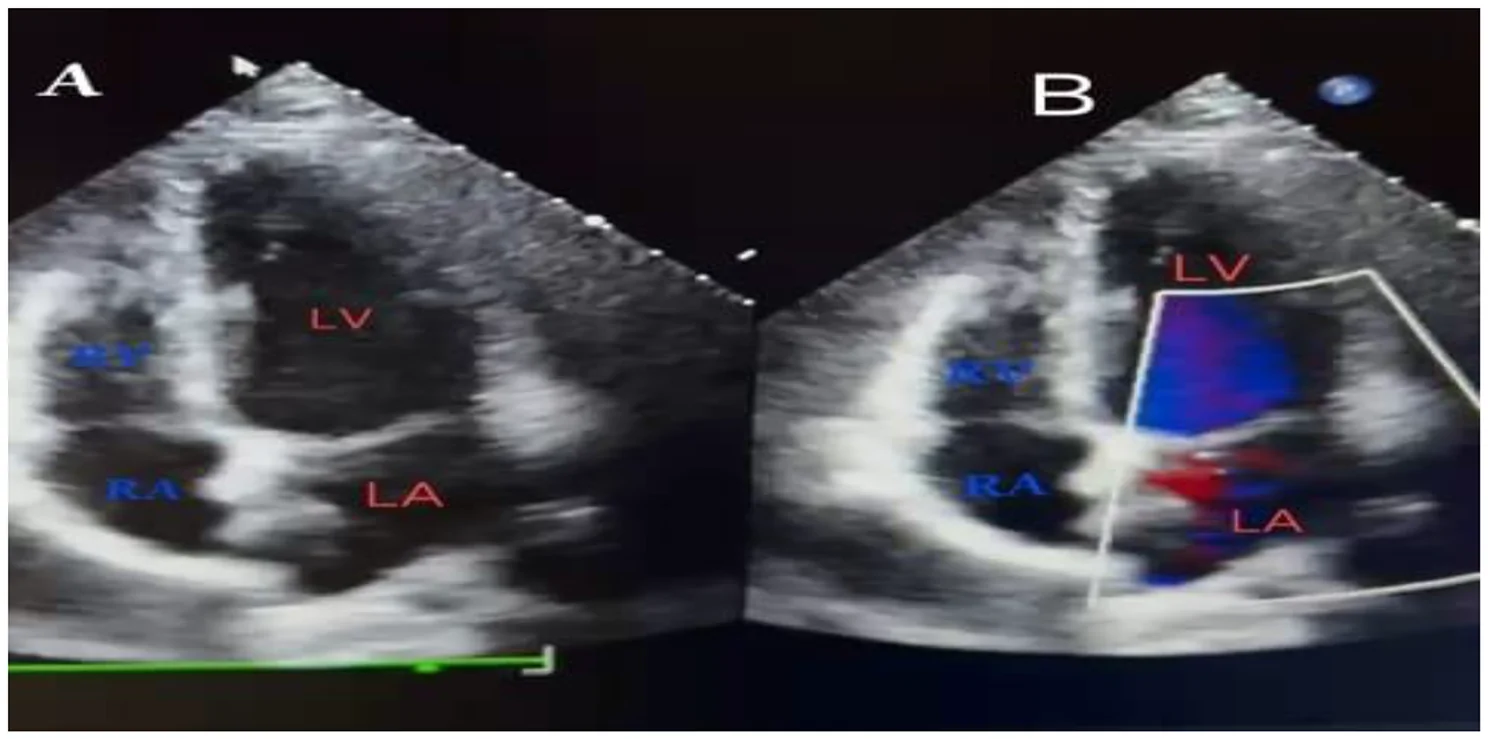
Transthoracic echocardiographic assessment following postoperative ST-segment elevation. (**A**) Apical four-chamber view demonstrating preserved biventricular systolic function without regional wall motion abnormalities. (**B**) Color Doppler imaging demonstrating no significant mitral or tricuspid valvular regurgitation and no evidence of pericardial effusion

Transthoracic echocardiography demonstrated preserved left ventricular systolic function, with a left ventricular ejection fraction of approximately 55–60%, and no new regional wall motion abnormalities. There was no significant new valvular abnormality and no pericardial effusion (Fig. 2). Serial echocardiographic assessment remained stable.

**Fig. 3:**
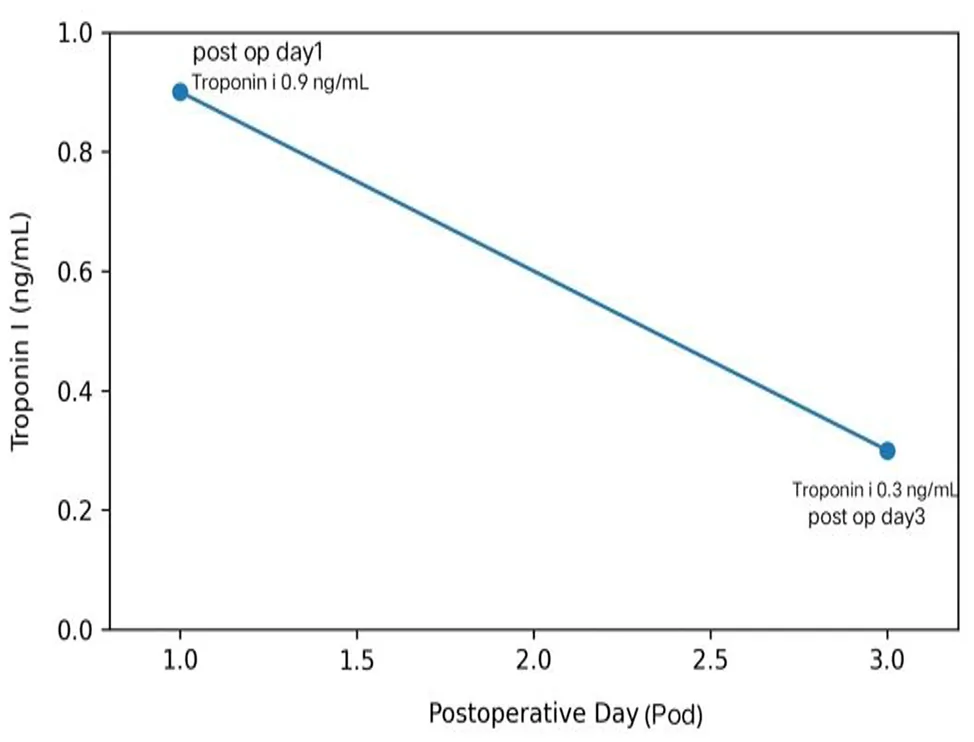
Serial cardiac troponin I measurements demonstrating a decline from **0.9 ng/mL** on postoperative day one to **0.3 ng/mL** on postoperative day three

High-sensitivity cardiac troponin I (hs-cTnI) was quantitatively measured using the routine clinical laboratory assay. The laboratory reference range was 0–0.6 ng/mL. A preoperative hs-cTnI measurement obtained approximately one month before surgery was 0.01 ng/mL. On postoperative day one, hs-cTnI was 0.9 ng/mL and decreased to 0.3 ng/mL by postoperative day three (Fig. 3).

The postoperative hs-cTnI elevation was therefore considered a real but modest elevation above the laboratory upper reference limit. Its interpretation was based on the postoperative context, serial decline, ECG evolution, echocardiographic findings, and clinical course rather than being considered insignificant in isolation.

### Differential diagnosis and diagnostic reasoning

The development of inferior ST-segment elevation on the first postoperative day raised concern for several possible etiologies:

1. Acute graft occlusion or graft-related ischemia
2. Perioperative myocardial infarction
3. Coronary vasospasm
4. Postoperative pericardial inflammation/pericarditis

Acute graft-related ischemia was considered particularly relevant because the inferior coronary territory had been revascularized through the PDA graft. The localized distribution of ST-segment elevation in leads II, III, and aVF therefore required careful exclusion of an RCA/PDA-territory ischemic event.

However, several findings reduced the likelihood of clinically significant ongoing ischemia. The patient remained hemodynamically stable without chest pain or low cardiac output syndrome. There were no reciprocal anterior ST-segment changes. Echocardiography repeatedly demonstrated preserved left ventricular systolic function without new regional wall motion abnormalities. The hs-cTnI level, although elevated above the laboratory reference range, showed a declining rather than progressive pattern.

In addition, PR-segment depression and a documented pericardial friction rub provided clinical and electrocardiographic evidence supporting pericardial inflammation. The elevated CRP and ESR were compatible with an inflammatory response, although these findings were recognized as nonspecific in the immediate postoperative period. The spontaneous resolution of the ST-segment abnormalities by postoperative day three further supported a transient inflammatory process.

Thus, the ECG distribution alone was not used to diagnose pericarditis. Rather, the overall clinical picture—including PR-segment depression, pericardial friction rub, clinical stability, preserved ventricular function, absence of new regional wall motion abnormalities, declining hs-cTnI, elevated inflammatory markers, and spontaneous ECG resolution—favored an early postoperative inflammatory process.

### Coronary angiography decision

Coronary angiography was considered because early graft failure, anastomotic complications, or perioperative myocardial infarction could not be excluded solely on the basis of non-invasive assessment. However, immediate invasive evaluation was deferred because the patient remained clinically stable, had preserved ventricular function, no new regional wall motion abnormality, no progressive biomarker elevation, and spontaneous improvement of the ECG abnormality.The decision was additionally influenced by the patient’s chronic ITP and postoperative platelet count, with invasive coronary assessment potentially requiring additional antithrombotic therapy and exposing the patient to increased bleeding risk. The decision to defer angiography therefore represented an individualized risk-benefit assessment rather than a conclusion that graft failure had been anatomically excluded.

### Management

The patient was managed conservatively with close clinical, electrocardiographic, and echocardiographic monitoring. Aspirin 75 mg once daily was continued as postoperative antiplatelet therapy. Prophylactic low-molecular-weight heparin was administered for venous thromboembolism prophylaxis rather than therapeutic anticoagulation. Indomethacin was administered for analgesic and anti-inflammatory treatment in view of the suspected postoperative pericardial inflammatory component. Intravenous nicorandil was administered empirically as an anti-ischemic measure during the period in which coronary ischemia and coronary vasospasm remained part of the differential diagnosis. Its use was not considered diagnostic evidence of myocardial ischemia or coronary vasospasm.

The patient’s platelet counts were closely monitored because of the underlying ITP and concurrent antithrombotic therapy. No clinically significant bleeding or thromboembolic complication occurred during hospitalization.

### Follow-up and outcome

**Fig. 4:**
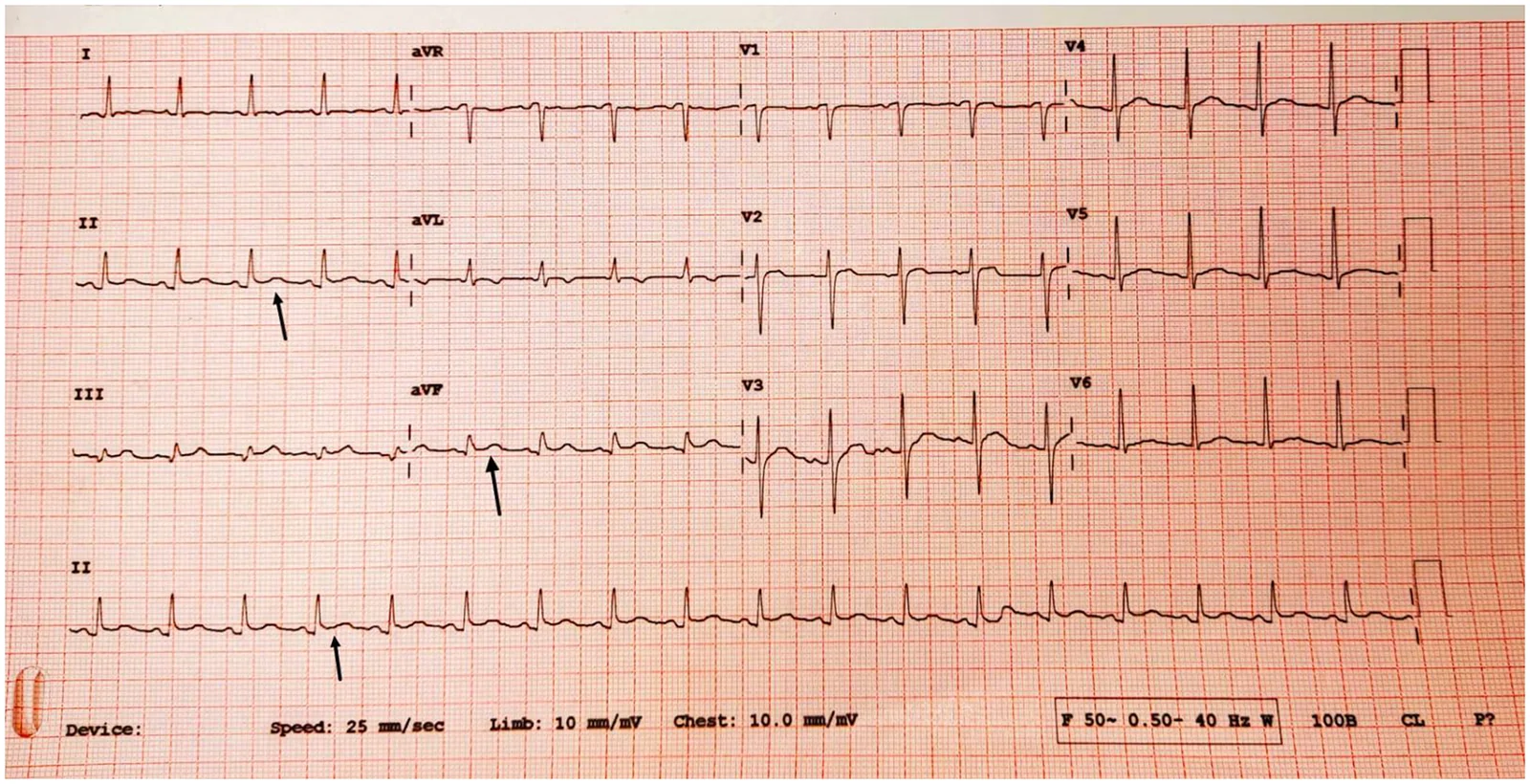
Twelve-lead electrocardiogram obtained on postoperative day three demonstrating sinus rhythm with complete resolution of the previously observed inferior ST-segment elevation (arrows)

The inferior ST-segment elevation progressively resolved and was no longer present on the postoperative day three ECG (Fig. 4).

The patient remained hemodynamically stable, with no recurrence of chest pain, no evidence of ventricular dysfunction, and no clinical evidence of ongoing graft-related ischemia. The hs-cTnI concentration declined from 0.9 ng/mL on postoperative day one to 0.3 ng/mL on postoperative day three. The platelet count remained clinically acceptable during the postoperative period.The patient was discharged on postoperative day seven in stable condition with appropriate medical therapy and follow-up recommendations.

At one-month follow-up, the patient remained asymptomatic. Electrocardiography showed normal sinus rhythm without recurrent ST-segment abnormalities. No bleeding or thrombotic complications were reported.

The chronological sequence of the patient’s clinical presentation, perioperative management, postoperative investigations, clinical decision-making, and outcome is summarized in (Table 1).

**Table 1:** Timeline of the patient’s clinical course, including preoperative assessment, perioperative management, postoperative investigations, clinical decision-making, and follow-up after coronary artery bypass grafting

Time point	Clinical event
Preoperative assessment	47-year-old man with chronic ITP and triple-vessel coronary artery disease; platelet count 57 × 10⁹/L
48 h before CABG	Romiplostim 250 µg administered subcutaneously for platelet optimization
48 h after romiplostim	Platelet count increased to 125 × 10⁹/L
Day 0	Four-vessel CABG performed; pedicled LIMA to LAD and RIMA Y-graft from LIMA to diagonal, ramus intermedius, and PDA
Intraoperative course	Initially attempted off-pump; converted to CPB because of deeply intramyocardial LAD and difficult distal PDA/PLV target exposure
Postoperative day 1	Inferior ST-segment elevation in II, III, and aVF with PR-segment depression; patient asymptomatic and hemodynamically stable
Postoperative day 1	Pericardial friction rub documented; echocardiography showed preserved LVEF without new regional wall motion abnormality or pericardial effusion
Postoperative day 1	hs-cTnI 0.9 ng/mL; CRP 120 mg/L; ESR 68 mm/h; platelet count 78 × 10⁹/L
Postoperative day 2	Platelet count 75 × 10⁹/L
Postoperative day 3	hs-cTnI decreased to 0.3 ng/mL; platelet count 81 × 10⁹/L; ST-segment elevation resolved
Postoperative day 4	Platelet count 85 × 10⁹/L
Postoperative day 7	Patient discharged in stable condition
One-month follow-up	Patient asymptomatic; no recurrent ECG abnormality, bleeding, or thrombotic complication

## Discussion

ST-segment elevation following CABG represents an important postoperative diagnostic challenge because it may be associated with perioperative myocardial infarction, early graft failure, or other forms of acute coronary compromise [1]. In the present case, the inferior distribution of ST-segment elevation was particularly relevant because the PDA territory had been revascularized using the RIMA Y-graft configuration. Therefore, an ischemic event involving the PDA graft or RCA territory had to be considered before a non-ischemic explanation could be accepted.

The interpretation of postoperative ST-segment elevation requires integration of the ECG with the clinical course, cardiac biomarkers, echocardiography, and, when appropriate, coronary angiography. Current revascularization guidance emphasizes comprehensive assessment when postoperative myocardial ischemia or infarction is suspected rather than reliance on an isolated ECG abnormality [2]. In the present patient, the absence of reciprocal anterior ST-segment depression, hemodynamic instability, ventricular dysfunction, or new regional wall motion abnormalities reduced the likelihood of a large ongoing ischemic event.

The hs-cTnI findings also required careful interpretation. The patient’s preoperative hs-cTnI was 0.01 ng/mL, increasing to 0.9 ng/mL on postoperative day one and subsequently declining to 0.3 ng/mL on postoperative day three. Thus, there was a genuine postoperative troponin elevation above the laboratory reference range, and it should not be described as insignificant. However, the declining trajectory, when considered together with the preserved ventricular function and absence of new regional wall motion abnormalities, was not suggestive of progressive myocardial injury. Cardiac biomarker elevation after CABG must be interpreted in the context of the surgical procedure and other objective evidence of myocardial ischemia [2].

An important aspect of this case was the presence of PR-segment depression and a documented pericardial friction rub. These findings provided additional evidence for pericardial inflammation and were particularly useful because inferior ST-segment elevation alone could not reliably distinguish an inflammatory process from RCA/PDA-territory ischemia. The diagnosis of pericarditis is based on an integrated clinical assessment, with characteristic ECG changes, pericardial friction rub, chest pain, and pericardial effusion representing potential diagnostic features [3, 7]. In this patient, no pericardial effusion was identified; therefore, the diagnosis was supported primarily by the combination of ECG findings and clinical examination together with the postoperative inflammatory context.

The markedly elevated CRP and ESR further supported the presence of postoperative inflammation. However, these markers are nonspecific following major cardiac surgery and cardiopulmonary bypass. Because preoperative CRP and ESR measurements had not been obtained, the magnitude of the postoperative inflammatory change relative to baseline could not be established. Accordingly, inflammatory markers were considered supportive rather than diagnostic evidence of pericarditis.

The patient’s lack of chest pain was also interpreted cautiously. Post-sternotomy analgesia may attenuate chest discomfort, meaning that absence of pain alone cannot exclude myocardial ischemia or pericardial inflammation. The diagnosis in this case therefore relied on the overall objective assessment rather than symptoms alone.

The decision to defer coronary angiography was another important component of management. Persistent ischemic symptoms, hemodynamic compromise, ventricular dysfunction, progressive cardiac biomarker elevation, or other evidence of acute coronary compromise would have strengthened the indication for urgent invasive evaluation [1, 2]. In contrast, this patient remained clinically stable, had preserved ventricular function without new regional wall motion abnormalities, demonstrated a declining hs-cTnI pattern, and showed spontaneous resolution of the ECG changes.

The presence of ITP added a further dimension to this risk-benefit assessment. In patients with thrombocytopenia requiring invasive cardiac procedures, the potential need for antiplatelet and anticoagulant therapy must be balanced against bleeding risk [4]. This consideration did not make angiography contraindicated, but it contributed to the decision that immediate invasive assessment was not justified in the setting of reassuring serial clinical and non-invasive findings.

Romiplostim was administered 48 h before surgery to increase the platelet count from 57 × 10⁹/L to 125 × 10⁹/L and thereby facilitate safe surgical revascularization. TPO-RAs are effective in increasing platelet counts in chronic ITP, but thromboembolic complications remain an important consideration, particularly in patients with additional cardiovascular risk factors [5, 6]. Therefore, romiplostim was considered part of the patient’s overall perioperative thrombotic-risk profile.

Importantly, the temporal association between romiplostim administration and postoperative ST-segment elevation does not establish causality. The patient had significant underlying coronary artery disease, had undergone major cardiac surgery with cardiopulmonary bypass, and experienced the expected postoperative inflammatory and prothrombotic environment. No objective evidence of acute graft occlusion, progressive myocardial injury, or systemic thromboembolism was identified. Accordingly, the present case does not support attributing the transient ST-segment elevation directly to romiplostim.

Post-CABG antithrombotic management also required individualized consideration. Aspirin was used as standard postoperative antiplatelet therapy, while LMWH was administered at prophylactic rather than therapeutic intensity for venous thromboembolism prevention. Current consensus recommendations emphasize the importance of balancing graft protection and thrombotic prevention against bleeding risk after CABG [8]. In this patient with ITP, platelet monitoring and clinical surveillance were particularly important.

The administration of indomethacin was intended for analgesic and anti-inflammatory treatment in the setting of suspected postoperative pericardial inflammation. It should not be interpreted as evidence that pericarditis had been established before treatment. Similarly, intravenous nicorandil was administered empirically while ischemia and coronary vasospasm remained part of the differential diagnosis. The use of an anti-ischemic agent therefore represented a precautionary therapeutic measure rather than confirmation of an ischemic mechanism.

Long-term real-world data support the effectiveness of romiplostim in chronic ITP while emphasizing the need for ongoing monitoring for potential adverse events, including thrombotic complications [9]. In a patient undergoing CABG, the presence of established coronary artery disease means that this risk must be considered in conjunction with the intrinsic thrombotic and inflammatory risks associated with major cardiac surgery.

Acute coronary syndrome guidelines emphasize timely recognition and management of suspected myocardial ischemia, particularly when there is persistent clinical or objective evidence of coronary compromise [10]. The present case illustrates that this principle must be applied together with individualized assessment of procedural risk. The decision to defer angiography was not based on the assumption that ischemic complications were impossible; rather, it reflected the absence of progressive objective evidence of ischemia and the competing bleeding risk associated with ITP.

The most important limitation of the diagnostic reasoning is that coronary angiography was not performed after the postoperative ST-segment elevation. Consequently, definitive anatomical confirmation of graft patency was not obtained. Although the patient’s clinical course strongly favored a transient inflammatory process, graft occlusion or a transient ischemic event cannot be completely excluded. The spontaneous resolution of the ECG abnormality, preserved ventricular function, absence of new regional wall motion abnormalities, and declining hs-cTnI made clinically significant ongoing graft-related ischemia less likely, but did not anatomically prove graft patency.

### Literature gap and novelty

Postoperative ST-segment elevation following CABG has primarily been discussed in relation to potentially serious ischemic complications, including perioperative myocardial infarction and early graft failure [1, 2]. Less information is available regarding the evaluation of transient postoperative ST-segment elevation in patients with significant hematological bleeding risk.The combination of CABG, chronic ITP, and preoperative romiplostim therapy creates a particularly complex clinical scenario in which ischemic, inflammatory, bleeding, and thrombotic risks must be considered simultaneously. The presence of ITP may alter the threshold for invasive investigation, while romiplostim introduces an additional consideration regarding thrombotic risk [4–6].

The novelty of this case lies not in establishing romiplostim as a cause of postoperative ST-segment elevation, but in demonstrating the structured evaluation of a transient inferior ST-segment abnormality in a patient with competing ischemic, bleeding, and thrombotic risks. The case emphasizes that postoperative ST-segment elevation should be interpreted using the complete clinical picture rather than the ECG distribution alone.

## Strengths and limitations

The principal strength of this case is the detailed assessment of a clinically challenging postoperative ECG abnormality in a patient with chronic ITP who received preoperative romiplostim. The evaluation incorporated serial ECGs, cardiac biomarkers, echocardiography, inflammatory markers, platelet monitoring, clinical examination, and assessment of the potential risks associated with invasive coronary angiography.

The principal limitation is the absence of postoperative coronary angiography or another direct anatomical assessment of graft patency. Therefore, acute graft failure or a transient ischemic event cannot be completely excluded. The decision to defer angiography was based on an individualized risk-benefit assessment in the setting of clinical stability, preserved ventricular function, absence of new regional wall motion abnormalities, declining hs-cTnI, spontaneous ECG resolution, and underlying ITP with increased bleeding risk. A further limitation is that preoperative CRP and ESR values were not available, preventing direct comparison with baseline inflammatory status. In addition, this is a single case report, and the observations cannot be generalized to all patients with ITP undergoing CABG or to all patients receiving romiplostim.

## Conclusion

Transient ST-segment elevation after CABG requires careful evaluation because ischemic and non-ischemic postoperative causes may present with overlapping electrocardiographic findings. In this patient with ITP who received preoperative romiplostim, the presence of PR-segment depression and a pericardial friction rub, together with preserved ventricular function, absence of new regional wall motion abnormalities, declining hs-cTnI, and spontaneous resolution of the ST-segment changes, was more consistent with a transient postoperative inflammatory process. The decision to defer coronary angiography was based on the overall clinical and objective assessment, while taking into account the patient’s competing bleeding and thrombotic risks.

Romiplostim should be regarded as part of the patient’s perioperative thrombotic-risk context rather than as a demonstrated cause of the postoperative ECG changes. Because coronary angiography was not performed, graft patency and an ischemic mechanism could not be definitively excluded. This case highlights the importance of integrating serial ECGs, cardiac biomarkers, echocardiography, clinical examination, surgical anatomy, and individual bleeding and thrombotic risk when evaluating postoperative ST-segment elevation after CABG.

## Data Availability

No datasets were generated or analysed during the current study.

## Abbreviations

CABG: Coronary Artery Bypass Grafting
ITP: Immune Thrombocytopenia
PMI: Perioperative Myocardial Infarction
TPO-RA: Thrombopoietin Receptor Agonist
ECG: Electrocardiogram
LVEF: Left Ventricular Ejection Fraction
LV: Left Ventricle
CRP: C-Reactive Protein
ESR: Erythrocyte Sedimentation Rate
hs-cTnI: High-Sensitivity Cardiac Troponin I
ACS: Acute Coronary Syndrome
PCI: Percutaneous Coronary Intervention
LMWH: Low-Molecular-Weight Heparin
CPB: Cardiopulmonary Bypass
TPO: Thrombopoietin
ACC: American College of Cardiology
AHA: American Heart Association
SCAI: Society for Cardiovascular Angiography and Interventions
ESC: European Society of Cardiology
EACTS: European Association for Cardio-Thoracic Surgery
CCS: Canadian Cardiovascular Society
LIMA: Left Internal Mammary Artery
RIMA: Right Internal Mammary Artery
LAD: Left Anterior Descending Artery
PDA: Posterior Descending Artery
PLV: Posterolateral Ventricular Artery
RCA: Right Coronary Artery
